# Supplementary material for: AcrIF11 is a potent CRISPR-specific ADP-ribosyltransferase encoded by phage and plasmid
Source: bioRxiv. 2025 Jun 2:2024.08.26.609590. Originally published 2024 Aug 26. Preprint. [Version 2] doi: 10.1101/2024.08.26.609590 (PMC11383003; doi:10.1101/2024.08.26.609590)

bioRxiv preprint doi: https://doi.org/10.1101/2024.08.26.609590; this version posted June 2, 2025. The copyright holder for this preprint (which was not certified by peer review) is the author/funder, who has granted bioRxiv a license to display the preprint in perpetuity. It is made available under aCC-BY 4.0 International license.

Supplementary Information

Supplementary Table 1. NMR assignment, structure calculation and validation statistics

**Degree of assignment**[a]	
Backbone (N and H^N) (%)	89.8
Side-chain H (%)	72.4
Side-chain non-H (%)	56.9
**Number of restraints**[b]	
NOE restraints	
Intra-residue (|i-j| = 0)	591
Sequential (|i-j| = 1)	545
Medium range (1 < |i-j| < 5)	423
Long range (|i-j| ≥ 5)	537
Ambiguous[a]	640
Total	2069
H-bond restraints	54
Long range (|i - j| ≥ 5)	40
Dihedral angle restraints (φ/ψ)	150/150
**Restraint statistics**[c]	
r.m.s. of NOE violations (Å)	0.525 ± 0.498
r.m.s. of dihedral violations (°)	2.60 ± 2.488
**r.m.s. from idealised covalent geometry**[d]	
Bonds (Å)	0.0042 ± 0.0002
Angles (°)	0.63 ± 0.041
Impropers (°)	2.28 ± 0.24
Structural quality	
Ramachandran statistics[e/f]	
Most favoured regions (%)	84.4 / 90.2
Allowed regions (%)	14.9 / 8.5
Generously allowed regions (%)	0.7 / NA
Disallowed regions (%)	0.0/1.3
Verify3D Z-score[g]	-4.98

bioRxiv preprint doi: https://doi.org/10.1101/2024.08.26.609590; this version posted June 2, 2025. The copyright holder for this preprint (which was not certified by peer review) is the author/funder, who has granted bioRxiv a license to display the preprint in perpetuity. It is made available under aCC-BY 4.0 International license.

Prosa II Z-score[h]	-0.99
Procheck Z-score (φ/ψ)[e]	-1.46
Procheck Z-score (all)[e]	-3.02
MolProbity Z-score[f]	-5.86
No. of close contacts[i]	11
**Coordinates precision (rmsd)**[b]	
All backbone atoms (Å)	2.8 / 2.3
All heavy atoms (Å)	3.3 / 2.8

Values reported by: [a]CCPNMR 2.5.2[39]; [b]Protein Structure Validation Software suite 1.5 & [c]PDBStat 5.12[53]; [d]Crystallography and NMR system (CNS) 1.2[54]; [e]Procheck[55] & [f]MolProbity[56]. The structural validation programs used were as follows: [g]Verify3D[57], [h]Prosa II[58], [e]Procheck[55], [f]MolProbity[56] and [i]PDB validation software.

Supplementary Table 2. List of bacterial genera containing AcrIF11 homologs

Pseudomonas
Pseudoxanthomonas
Halopseudomonas
Xanthomonas
Pigmentiphaga
Marinobacterium
Enterobacter
Citrobacter
Klebsiella
Raoultella
Delftia
Paramixta
Dickeya
Cedecea
Yersinia
Pectobacterium
Brenneria
Serratia
Buttiauxella
Moraxella
Lelliottia
Escherichia
Erwinia
Actinobacillus
Haemophilus
Frischella
Rouxiella

bioRxiv preprint doi: https://doi.org/10.1101/2024.08.26.609590; this version posted June 2, 2025. The copyright holder for this preprint (which was not certified by peer review) is the author/funder, who has granted bioRxiv a license to display the preprint in perpetuity. It is made available under aCC-BY 4.0 International license.

Megasphaera
Billgrantia
Mitsuokella
Halomonas
Chromohalobacter
Sphaerochaeta
Desulfobulbus
Alcanivorax
Enterococcus
Photorhabdus

Supplementary Table 3. 1 L M9 Minimal Media Recipe for 15N labeled proteins

**Component**	**Amount**
10x M9 salts	100 mL
1M Magnesium sulphate	1 mL
1M Calcium chloride	100 uL
20% Thiamine	100 uL
0.003 g/mL Iron II sulphate heptahydrate	1 mL
10x MEM Vitamin mix	10 mL
20% Glucose	40 mL
15N Ammonium sulfate (15N source)	1 g
MilliQ H2O	860 mL

All solutions are dissolved in MilliQ H2O

Supplementary Table 4. 1 L M9 Minimal Media Recipe for 15N 13C labeled proteins

**Component**	**Amount**
10x M9 salts	100 mL
1M Magnesium sulphate	1 mL
1M Calcium chloride	100 uL

bioRxiv preprint doi: https://doi.org/10.1101/2024.08.26.609590; this version posted June 2, 2025. The copyright holder for this preprint (which was not certified by peer review) is the author/funder, who has granted bioRxiv a license to display the preprint in perpetuity. It is made available under aCC-BY 4.0 International license.

20% Thiamine	100 uL
Iron II sulphate heptahydrate	3 mg
10x MEM Vitamin mix	10 mL
10% 13C Glucose	40 mL
15N Ammonium sulfate	1 g
MilliQ H2O	860 mL

All solutions are dissolved in MilliQ H2O

Supplementary Figure 1. Sequence logo of AcrIF11 homologs.

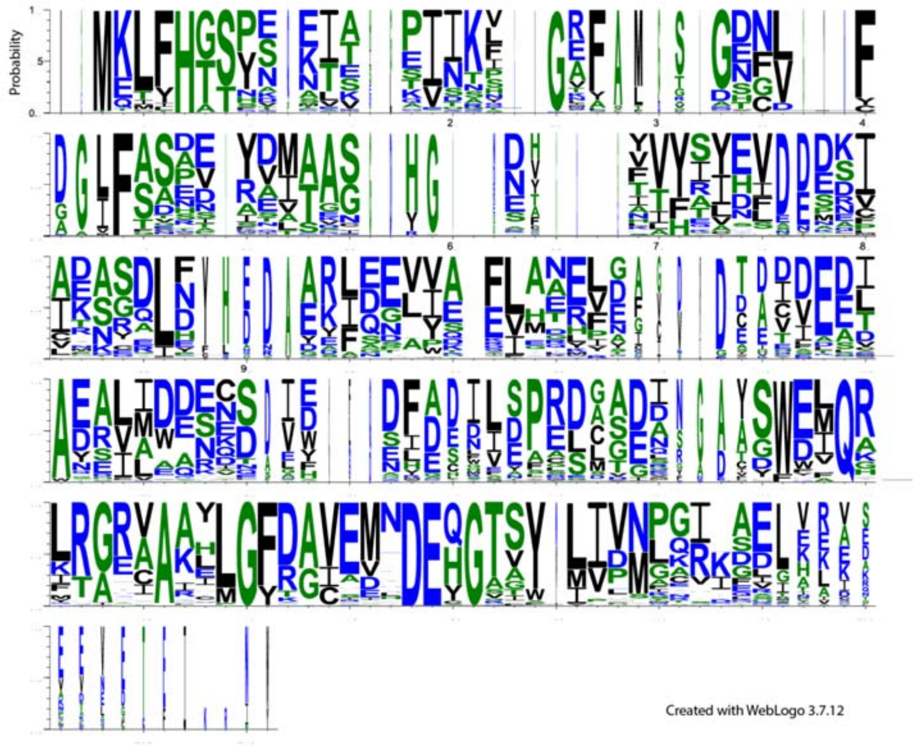

bioRxiv preprint doi: https://doi.org/10.1101/2024.08.26.609590; this version posted June 2, 2025. The copyright holder for this preprint (which was not certified by peer review) is the author/funder, who has granted bioRxiv a license to display the preprint in perpetuity. It is made available under aCC-BY 4.0 International license.

Supplementary Figure 2. Alignment of experimental and predicted structures of AcrIF11$_{Pae1}$ and AcrIF11$_{Pae2}$

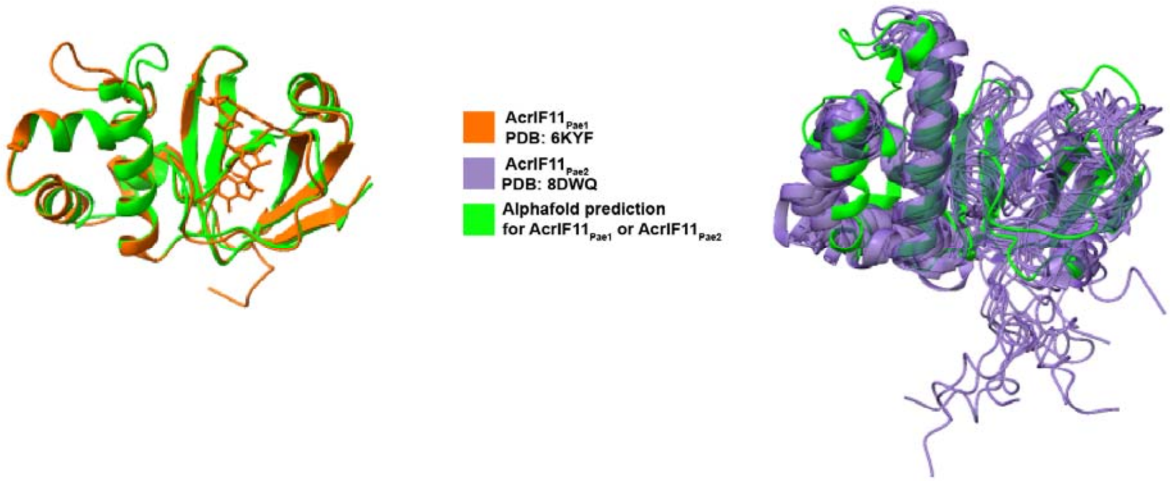

Supplementary Figure 3. Confidence of Alphafold2 predictions for AcrIF11 homologs.

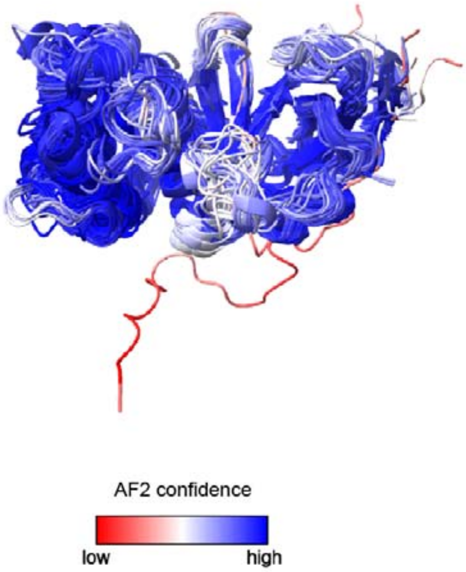

Supplementary Figure 4. Enlarged version of the AcrIF11 phylogeny in Figure 1F.

See supplementary file.

bioRxiv preprint doi: https://doi.org/10.1101/2024.08.26.609590; this version posted June 2, 2025. The copyright holder for this preprint (which was not certified by peer review) is the author/funder, who has granted bioRxiv a license to display the preprint in perpetuity. It is made available under aCC-BY 4.0 International license.

Supplementary Figure 5. AcrIF11 phylogeny sequence alignment

Below is a diverse sampling of the sequence alignment used to build the AcrIF11 phylogeny. Phylogeny construction is discussed in the Methods section.

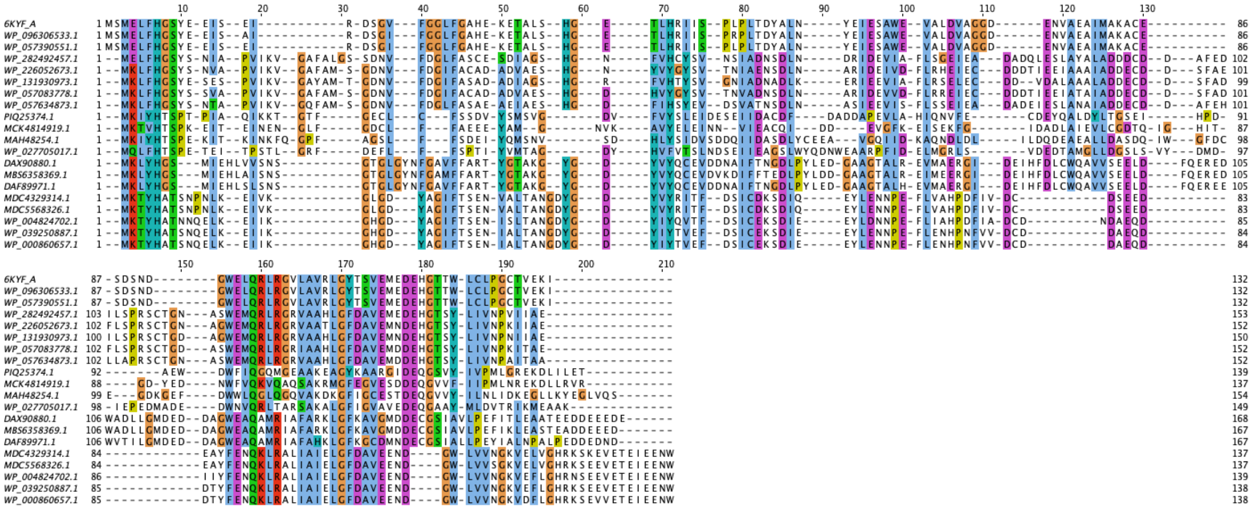

bioRxiv preprint doi: https://doi.org/10.1101/2024.08.26.609590; this version posted June 2, 2025. The copyright holder for this preprint (which was not certified by peer review) is the author/funder, who has granted bioRxiv a license to display the preprint in perpetuity. It is made available under aCC-BY 4.0 International license.

Supplementary Figure 6. Replicates of lysogen growth experiment

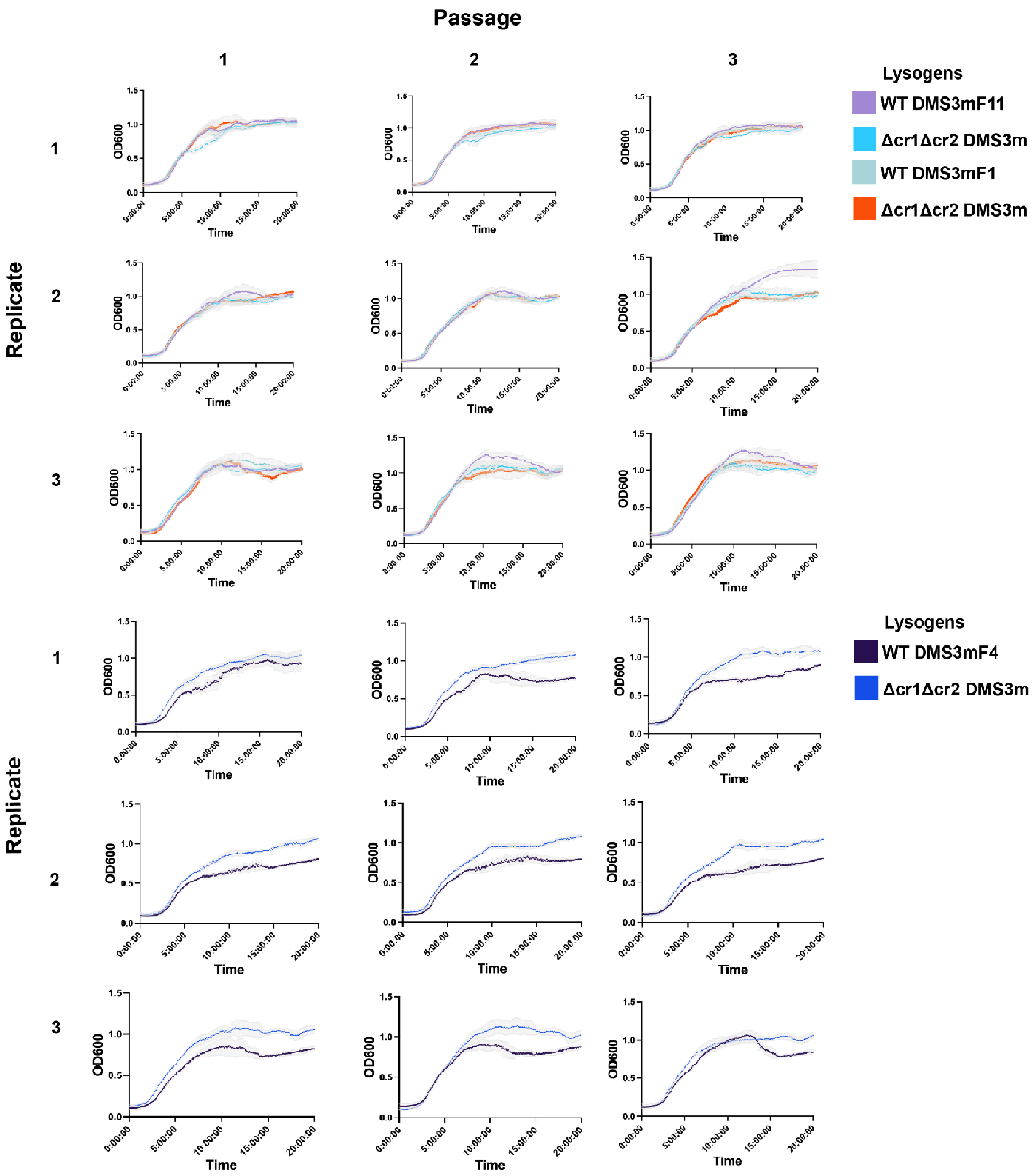

Supplementary Figure 7. Quantification of macrodomain lysate blot

bioRxiv preprint doi: https://doi.org/10.1101/2024.08.26.609590; this version posted June 2, 2025. The copyright holder for this preprint (which was not certified by peer review) is the author/funder, who has granted bioRxiv a license to display the preprint in perpetuity. It is made available under aCC-BY 4.0 International license.

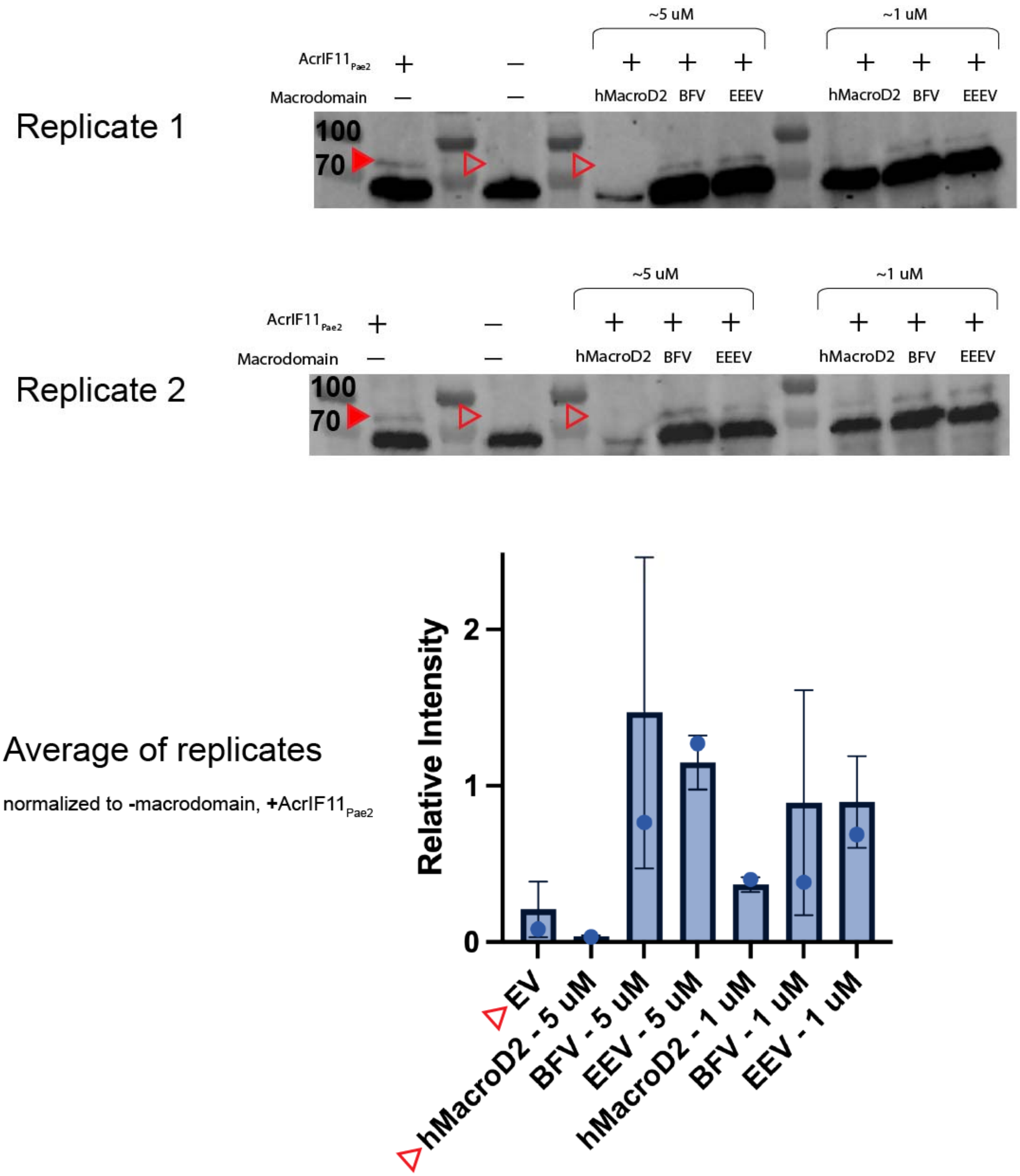

bioRxiv preprint doi: https://doi.org/10.1101/2024.08.26.609590; this version posted June 2, 2025. The copyright holder for this preprint (which was not certified by peer review) is the author/funder, who has granted bioRxiv a license to display the preprint in perpetuity. It is made available under aCC-BY 4.0 International license.

Supplementary Figure 8. Verification of macrodomain lysate blot loading via Ponceau

All labels are the same as in Fig 5B. Brown arrow indicates hMacroD2.

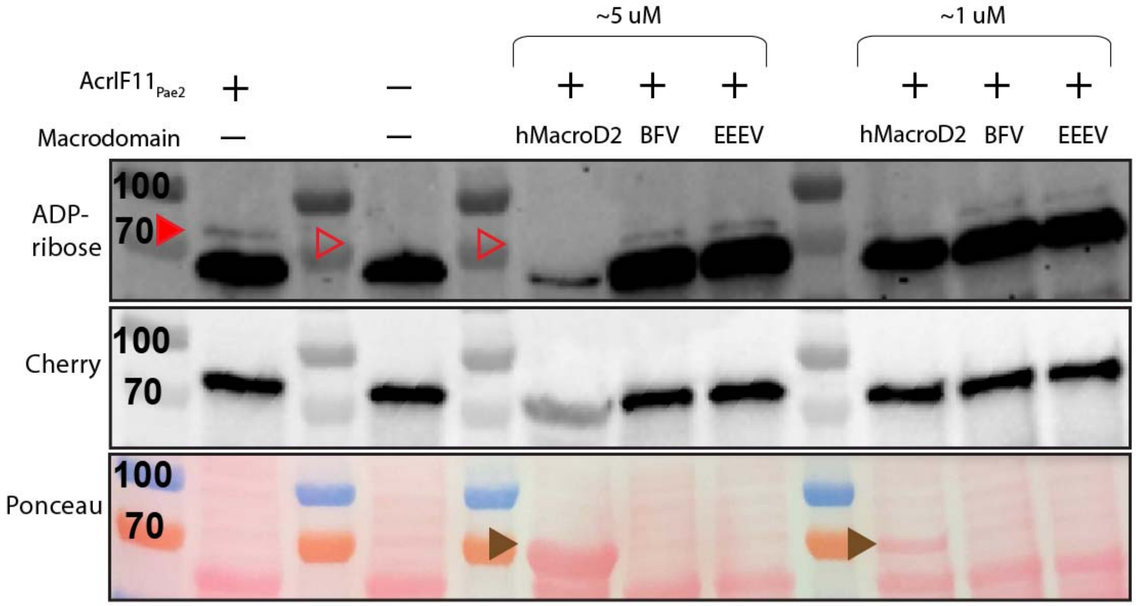

bioRxiv preprint doi: https://doi.org/10.1101/2024.08.26.609590; this version posted June 2, 2025. The copyright holder for this preprint (which was not certified by peer review) is the author/funder, who has granted bioRxiv a license to display the preprint in perpetuity. It is made available under aCC-BY 4.0 International license.

Supplementary Figure 9. Liquid growth curves of PA14 WT overexpressing non-endogenous macrodomains

PA14 WT was infected with DMS3mF11$_{Pae1}$vir, following the lytic infection protocol listed in the Methods section above.

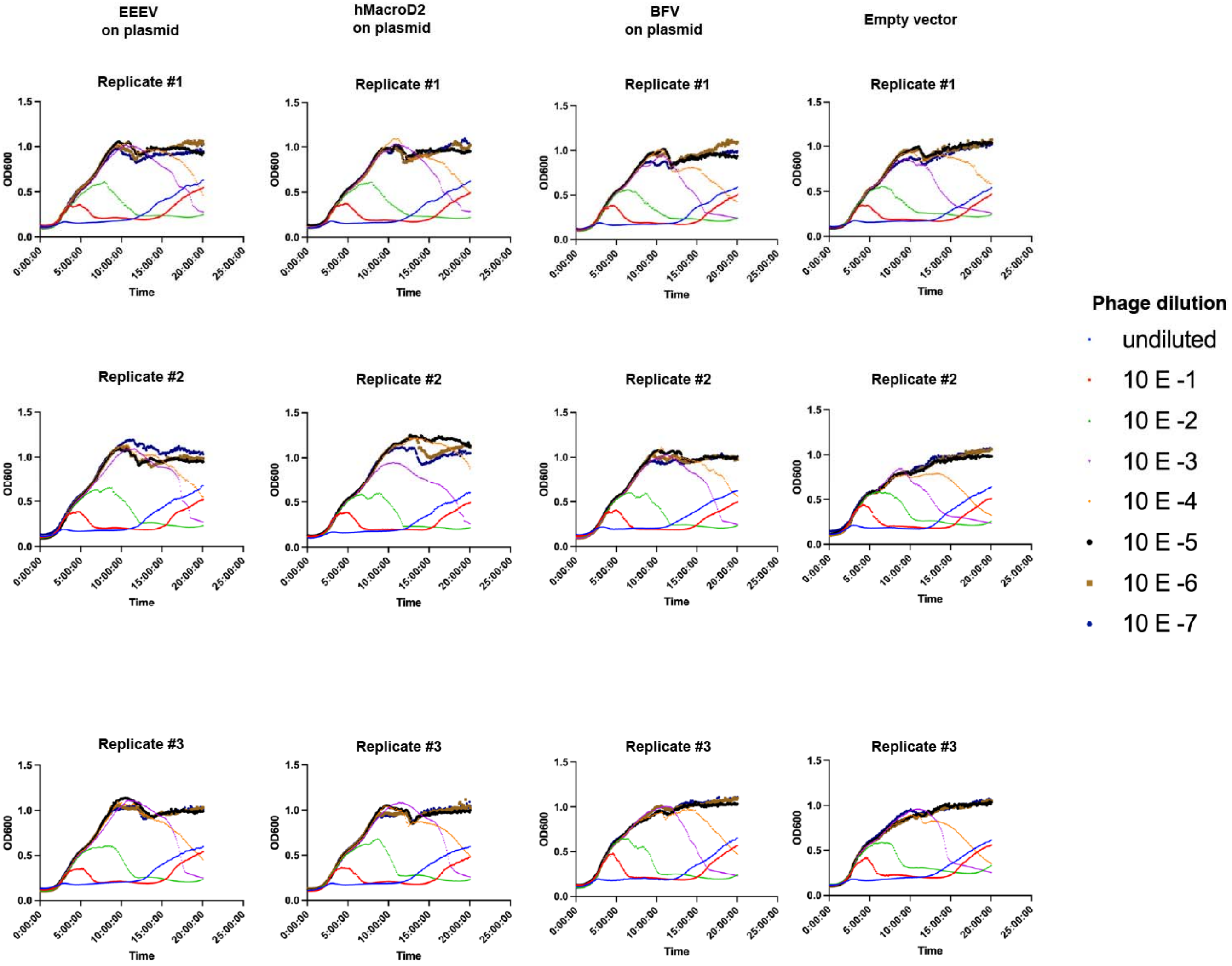

bioRxiv preprint doi: https://doi.org/10.1101/2024.08.26.609590; this version posted June 2, 2025. The copyright holder for this preprint (which was not certified by peer review) is the author/funder, who has granted bioRxiv a license to display the preprint in perpetuity. It is made available under aCC-BY 4.0 International license.

Supplementary Figure 10. Alphafold3 prediction of Csy complex + NAD + crRNA + AcrIF11$_{Pae2}$ colored by pLDDT

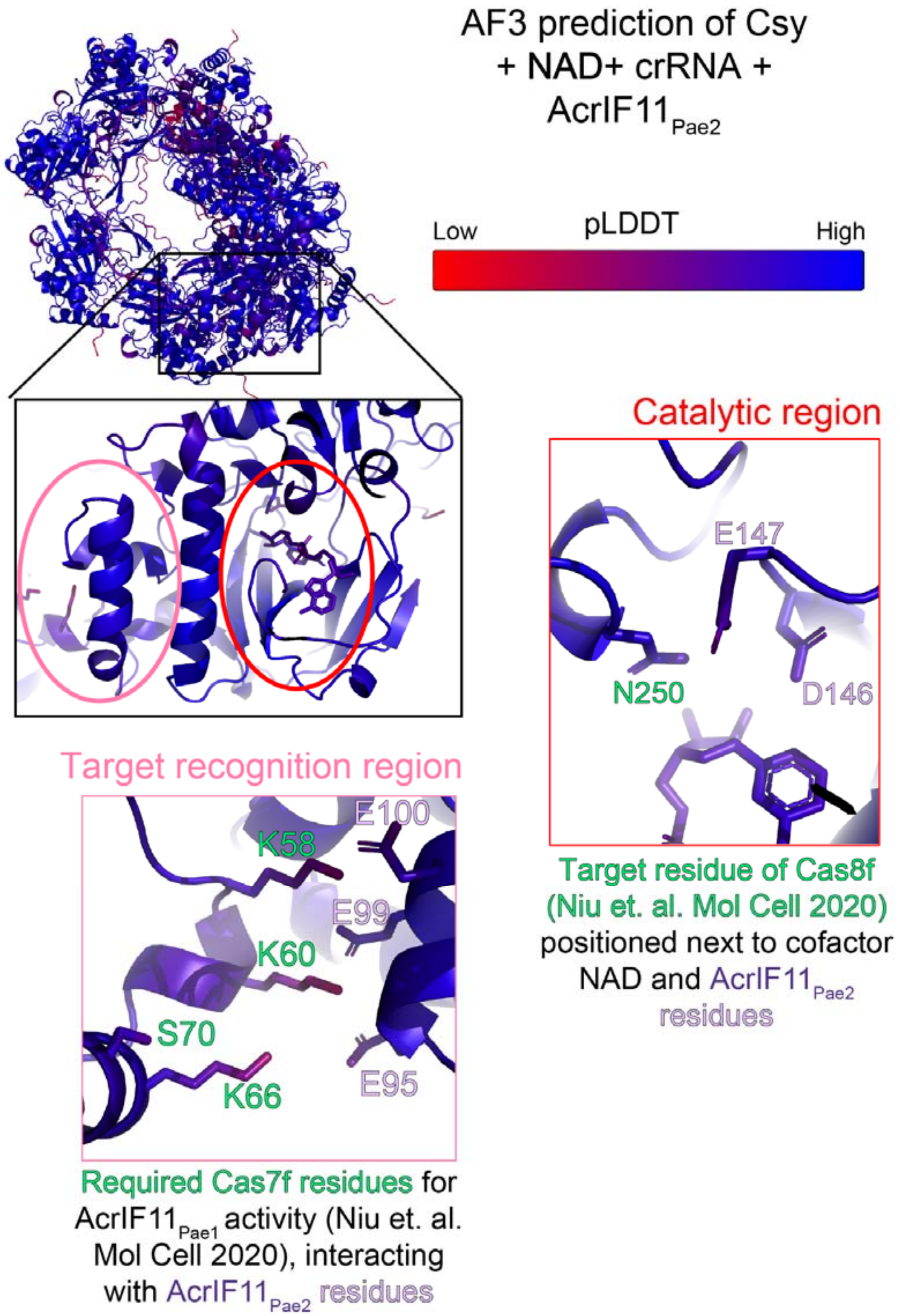

Supplement: 1 [file NIHPP2024.08.26.609590v2-supplement-1.pdf]
