# Supplementary material for: AcrIF11 is a potent CRISPR-specific ADP-ribosyltransferase encoded by phage and plasmid
Source: bioRxiv. 2024 Aug 26:2024.08.26.609590. Preprint. [Version 1] doi: 10.1101/2024.08.26.609590 (PMC11383003; doi:10.1101/2024.08.26.609590)
Supplement: Supplementary file 1 [file media-1.pdf]

Supplementary Information

Table 1. NMR assignment, structure calculation and validation statistics

**Degree of assignment**[a]	
Backbone (N and H^N) (%)	89.8
Side-chain H (%)	72.4
Side-chain non-H (%)	56.9
**Number of restraints**[b]	
NOE restraints	
Intra-residue (|i-j| = 0)	591
Sequential (|i-j| = 1)	545
Medium range (1 < |i-j| < 5)	423
Long range (|i-j| ≥ 5)	537
Ambiguous[a]	640
Total	2069
H-bond restraints	54
Long range (|i - j| ≥ 5)	40
Dihedral angle restraints (φ/ψ)	150/150
**Restraint statistics**[c]	
r.m.s. of NOE violations (Å)	0.525 ± 0.498
r.m.s. of dihedral violations (°)	2.60 ± 2.488
**r.m.s. from idealised covalent geometry**[d]	
Bonds (Å)	0.0042 ± 0.0002
Angles (°)	0.63 ± 0.041
Impropers (°)	2.28 ± 0.24
Structural quality	
Ramachandran statistics[e/f]	
Most favoured regions (%)	84.4 / 90.2
Allowed regions (%)	14.9 / 8.5
Generously allowed regions (%)	0.7 / NA
Disallowed regions (%)	0.0/1.3
Verify3D Z-score[g]	-4.98
Prosa II Z-score[h]	-0.99
Procheck Z-score (φ/ψ)[e]	-1.46

Procheck Z-score (all)[e]	-3.02
MolProbity Z-score[f]	-5.86
No. of close contacts[i]	11
**Coordinates precision (rmsd)**[b]	
All backbone atoms (Å)	2.8 / 2.3
All heavy atoms (Å)	3.3 / 2.8

Values reported by: [a]CCPNMR 2.5.2[30]; [b]Protein Structure Validation Software suite 1.5 & [c]PDBStat 5.12[44]; [d]Crystallography and NMR system (CNS) 1.2[45]; [e]Procheck[46] & [f]MolProbity[47]. The structural validation programs used were as follows: [g]Verify3D[48], [h]Prosa II[49], [e]Procheck[46], [f]MolProbity[47] and [i]PDB validation software.

Table 2. List of bacterial genera containing AcrIF11 homologs

Pseudomonas
Halopseudomonas
Xanthomonas
Enterobacter
Citrobacter
Pectobacterium
Yersinia
Klebsiella
Raoultella
Dickeya
Cedecea
Brenneria
Serratia
Moraxella
Escherichia
Lelliottia
Erwinia
Actinobacillus
Salmonella
Haemophilus
Frischella
Rouxiella
Shigella
Halomonas
Megasphaera
Chromohalobacter
Desulfobulbus
Alcanivorax
Enterococcus

Table 3. 1 L M9 Minimal Media Recipe for 15N labeled proteins

Component	Amount
10x M9 salts	100 mL
1M Magnesium sulphate	1 mL
1M Calcium chloride	100 uL
20% Thiamine	100 uL
0.003 g/mL Iron II sulphate heptahydrate	1 mL
10x MEM Vitamin mix	10 mL
20% Glucose	40 mL
15N Ammonium sulfate (15N source)	1 g
MilliQ H2O	860 mL

All solutions are dissolved in MilliQ H2O

Table 4. 1 L M9 Minimal Media Recipe for 15N 13C labeled proteins

Component	Amount
10x M9 salts	100 mL
1M Magnesium sulphate	1 mL
1M Calcium chloride	100 uL
20% Thiamine	100 uL
Iron II sulphate heptahydrate	3 mg
10x MEM Vitamin mix	10 mL
10% 13C Glucose	40 mL
15N Ammonium sulfate	1 g
MilliQ H2O	860 mL

All solutions are dissolved in MilliQ H2O

Figure 1. Sequence logo of AcrIF11 homologs

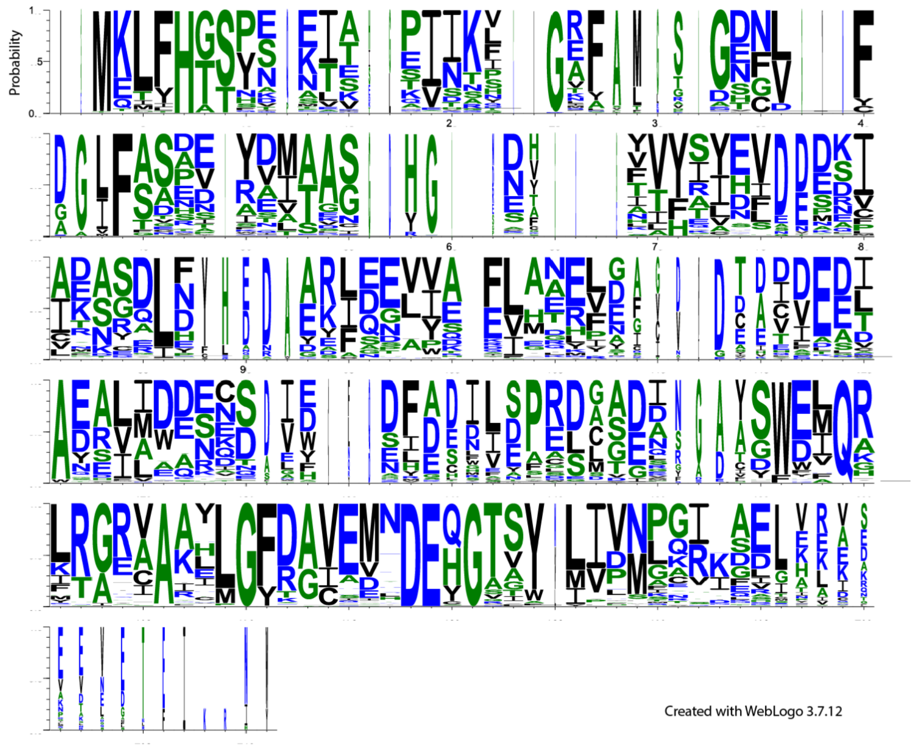

Figure 2. Alignment of experimental and predicted structures of $AcrIF11_{Pae1}$ and $AcrIF11_{Pae2}$

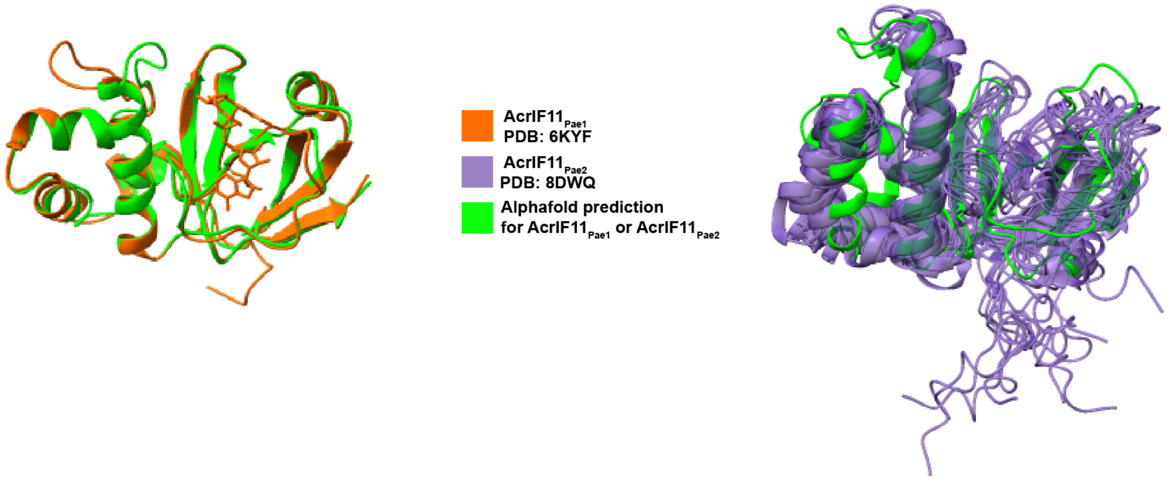

Figure 3. Confidence of Alphafold2 predictions for AcrIF11 homologs.

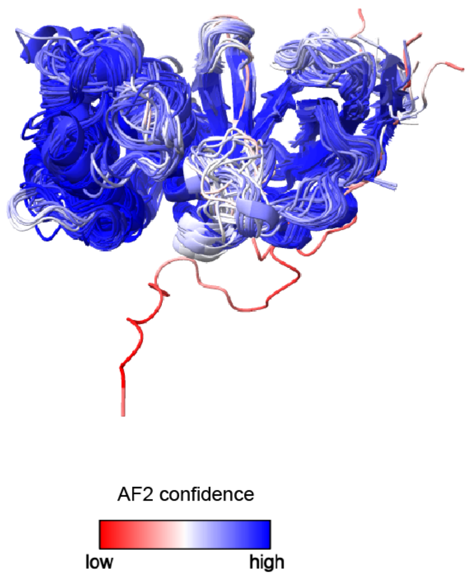

Figure 4. Liquid growth curves of PA14 WT overexpressing non-endogenous macrodomains

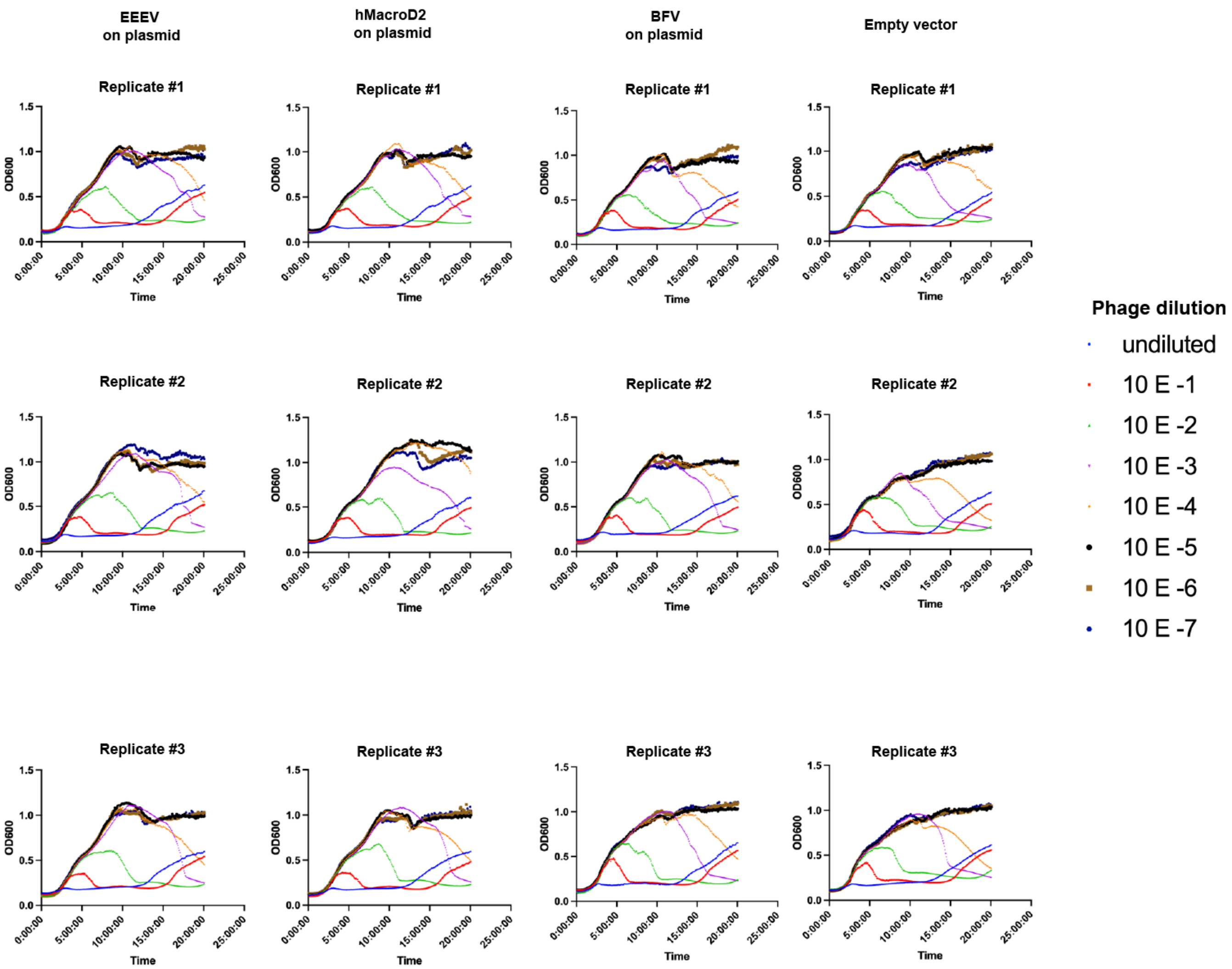

Figure 5. Alphafold3 prediction of Csy complex with AcrIF11

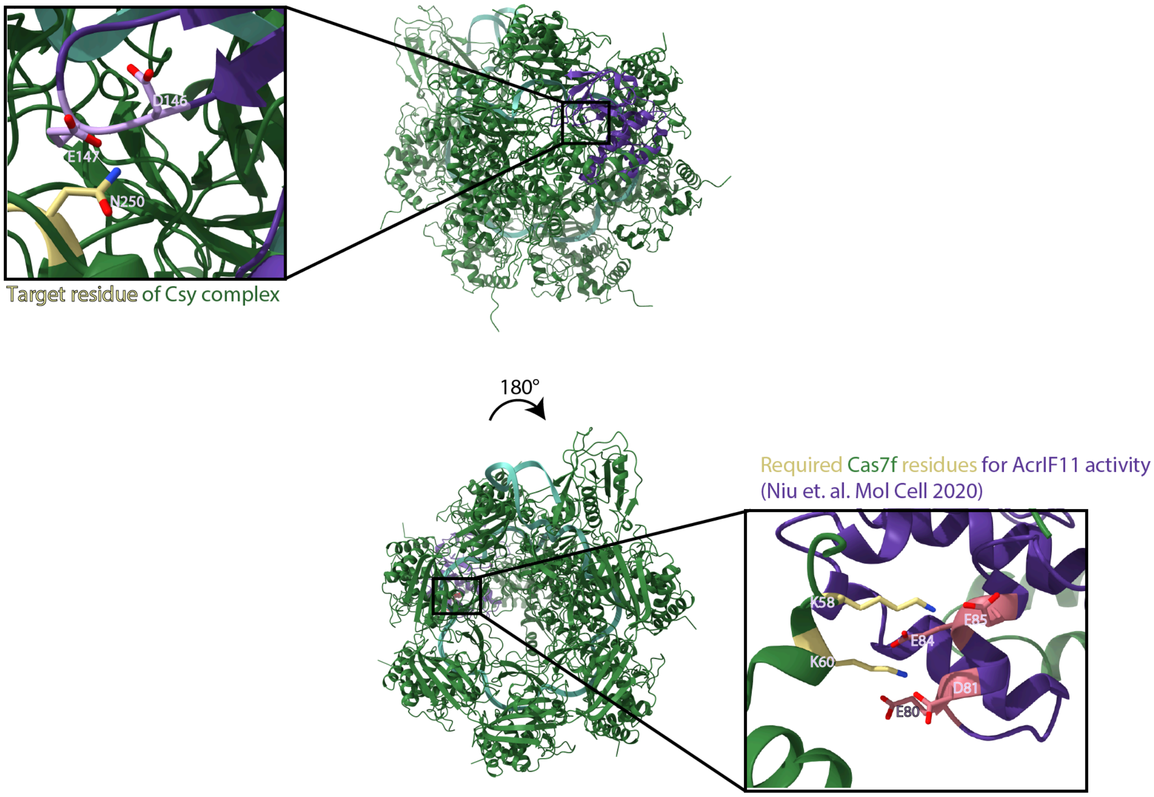

All five output models from Alphafold3 were aligned to confirm structural similarity, but only one model was displayed for the sake of clarity.
Scores of iPTM = 0.67 and pTM = 0.7 indicate this is a medium confidence prediction.[50 51]
